# Insights in Pharmaceutical Pollution: The Prospective Role of eDNA Metabarcoding

**DOI:** 10.3390/toxics11110903

**Published:** 2023-11-05

**Authors:** Charikleia Papaioannou, George Geladakis, Vasiliki Kommata, Costas Batargias, George Lagoumintzis

**Affiliations:** 1 Department of Biology, University of Patras, 26504 Patras, Greece; xpapaioannou@upatras.gr (C.P.); geladakisg@upatras.gr (G.G.); kommatav@upatras.gr (V.K.); 2 Department of Pharmacy, University of Patras, 26504 Patras, Greece

**Keywords:** aquatic pollution, biodiversity, biomonitoring, ecotoxicology, eDNA analysis, pharmaceutical active chemicals

## Abstract

Environmental pollution is a growing threat to natural ecosystems and one of the world’s most pressing concerns. The increasing worldwide use of pharmaceuticals has elevated their status as significant emerging contaminants. Pharmaceuticals enter aquatic environments through multiple pathways related to anthropogenic activity. Their high consumption, insufficient waste treatment, and the incapacity of organisms to completely metabolize them contribute to their accumulation in aquatic environments, posing a threat to all life forms. Various analytical methods have been used to quantify pharmaceuticals. Biotechnology advancements based on next-generation sequencing (NGS) techniques, like eDNA metabarcoding, have enabled the development of new methods for assessing and monitoring the ecotoxicological effects of pharmaceuticals. eDNA metabarcoding is a valuable biomonitoring tool for pharmaceutical pollution because it (a) provides an efficient method to assess and predict pollution status, (b) identifies pollution sources, (c) tracks changes in pharmaceutical pollution levels over time, (d) assesses the ecological impact of pharmaceutical pollution, (e) helps prioritize cleanup and mitigation efforts, and (f) offers insights into the diversity and composition of microbial and other bioindicator communities. This review highlights the issue of aquatic pharmaceutical pollution while emphasizing the importance of using modern NGS-based biomonitoring actions to assess its environmental effects more consistently and effectively.

## 1. Introduction

Human activities such as industrialization, urbanization, and economic development contribute synergistically to increased environmental pollution in aquatic habitats, such as rivers, lakes, and marine environments [1]. Chemical pollutants, such as heavy metals and industrial and pharmaceutical chemicals, can disrupt the balance of essential nutrients and oxygen levels, impair water quality, and make toxic or unsuitable conditions for aquatic life [2]. Water pollution can also harm biodiversity and disrupt photosynthesis in aquatic plants, significantly impacting ecosystems relying on these plants [3]. Both terrestrial and aquatic plants can absorb pollutants from water (as their main nutrient source) and transfer them through the food chain to animals and humans [4]. Pharmaceutical drugs and their metabolites contribute to water pollution by entering water bodies, disrupting the normal biological processes of aquatic organisms, and leading to the development of drug-resistant strains of bacteria [5]. In aquatic environments, including surface water, urban wastewater, wastewater treatment plants, groundwater, drinking water, and even seawater, the concentration range of predominant individual pharmaceutical compounds is typically observed to be between nanograms per liter (ng/L) to micrograms per liter (μg/L). Nevertheless, effluent from treatment plants that receive waste from pharmaceutical manufacturing facilities has been documented to contain concentrations as high as several milligrams per liter (mg/L) [6]. Furthermore, the distribution of pharmaceuticals in aquatic environments is geographically specific and contingent upon drug use patterns [7].

In recent decades, a major concern has arisen due to the increasing use of pharmaceutical products and their detrimental effects on the environment, wildlife, and humans [8]. Hignite and Azarnoff were the pioneering authors who initially documented the existence of pharmaceutical compounds in both wastewater and natural water during the late 1970s [9]. Since then, our comprehension of pharmaceuticals’ origins, fate, and ecotoxicity has advanced [10,11,12]. Pharmaceuticals are chemicals for diagnosing, preventing, and treating humans and animals [13]. They are vital to modern human and veterinary medicine, and their use is rising worldwide because of population increase, aging demographics, economic expansion, and the rising demand for animal protein in intensified food production [8,14]. Pharmaceuticals are one of the few chemical groups explicitly designed to act on living organisms. Pharmaceutical active chemicals (PhACs) are the biologically active components of pharmaceutical medications. These PhACs may be natural or synthetic chemical compounds typically found in therapeutic and veterinary medicines. 

Over the past twenty years, the negative effects of pharmaceutical products on the environment, wildlife, and humans have been recognized as a serious problem that must be addressed globally [8,15,16,17,18,19]. Slowly degradable or non-degradable PhACs pose a unique risk when they enter, remain, or disperse in the environment and are thus considered environmentally persistent pharmaceutical pollutants (EPPPs). The extensive consumption of numerous pharmaceutical products results in their subsequent release into the environment, making them serious emerging contaminants. [5,12,20]. Multiple mechanisms and pathways aid PhACs and their metabolites enter aquatic environments such as seas, rivers, and aquaculture facilities [21,22,23,24]. These pathways include the excessive use of pharmaceutical products like antibiotics, β-blockers, psychoactive substances, endocrine disruptors, analgesics, anticancer drugs, and non-steroidal anti-inflammatory drugs (NSAIDs), as well as processes such as oxidation, photolysis, wastewater treatment plants, pharmaceutical manufacturing, and improper medication disposal [6,19,25,26,27]. Additively, microplastics can also carry pharmaceutical elements and metabolites, increasing environmental exposure [28]. Due to their massive global consumption and the inability of organisms to completely metabolize drugs [29,30,31], pharmaceutical residues in aquatic environments and their long-term toxic effects on living organisms are becoming more of a concern [21,26,30,32,33].

According to the existing literature, antibiotics are the most frequently identified pharmaceuticals in aquatic environments, followed by non-steroidal anti-inflammatory drugs (NSAIDs) and psychotropic substances [34]. Antibiotics are chemical compounds that can eradicate or impede the proliferation of pathogens. Consequently, they have been extensively employed in the management, regulation, and prevention of infectious diseases in humans, animals, and plants [35,36]. Multiple antibiotics have been documented to exhibit high levels of toxicity towards various aquatic organisms, as indicated by toxicity unit values above 100 for acute toxicity and 1000 for chronic toxicity. Erythromycin exhibited the highest level of toxicity among the antibiotics, as indicated by its elevated acute and chronic toxicity unit values [37,38].

Analgesics and NSAIDs are PhACs that are extensively utilized on a global scale [39]. These substances are commonly prescribed for analgesic purposes in human medical treatment. However, they are also frequently available for purchase without a prescription, commonly referred to as “over-the-counter” medications. Certain NSAIDs may not elicit immediate physiological responses but instead exert long-term effects on specific organisms. As an example, Cleuvers [40] reported that naproxen exhibited an EC50 (half maximal effective concentration) value of 174 mg/L and a NOEC (no-observed-effect concentration) value of 0.15 mg/L for *Daphnia magna*. Based on studies conducted by Martins et al. [41] and Załeska-Radziwiłl et al. [42], ciprofloxacin exhibited an EC50 value of 65.3 mg/L for *Daphnia magna*, while the NOEC value was 0.156 mg/L. 

Psychiatric medications are pharmacological agents that possess psychoactive properties, influencing the internal neurochemical processes of the brain and the central nervous system. Therefore, these pharmaceuticals manage mental and neurological disorders [43,44]. In aquatic environments, the most frequently identified psychiatric pharmaceuticals include antidepressants, anxiolytics, and antiepileptic drugs (AEDs). According to Duarte et al. [45], the administration of fluoxetine, a psychiatric medication, resulted in significant DNA damage in meagre (*Argyrosomus regius*) when exposed to a concentration of 3 μg/L, as compared to the control group. Additionally, Aguirre-Martínez et al. [46] emphasized the significant DNA damage caused by carbamazepine, a psychiatric medication, to *Corbicula fluminea*. Notably, even at the lowest dose examined (0.1 μg/L) and after an exposure period of 21 days, carbamazepine had a considerable impact on DNA integrity.

Pharmaceuticals exhibit significant diversity in their physicochemical qualities, resulting in a wide range of biological variances. The water solubility, hydrophobicity, volatility, and other similar properties of substances can significantly influence their actions and ultimate destiny within aquatic ecosystems. The fate of pharmaceuticals is influenced by various factors, including dissociation constants (pKa), solid–water distribution coefficients (Kd), organic carbon-based sorption coefficients (log Koc), and octanol-water partition coefficients (Kow). These factors play a role in determining the extent of sorption, partitioning, hydrolysis, photodegradation, and biodegradation processes [47,48,49]. Furthermore, it should be noted that numerous pharmaceuticals possess acidic and/or basic functional groups, hence allowing for the existence of anionic, cationic, neutral, or zwitterionic forms under varying pH values [50]. The variability of these factors is contingent upon the pKa and Kow values of the molecule, as stated by Patel et al. [6]. The significance of chirality in relation to the environmental destiny of pharmaceuticals is noteworthy, because approximately 50% of pharmaceutical products are marketed and distributed as individual enantiomers [51]. Enantioselective reactions involve the subjection of a certain enantiomer to distinct biotransformations compared to its enantiomeric counterpart [52].

To evaluate the environmental hazards associated with pharmaceuticals, it is imperative to consider many factors, such as the quantities in which they are used, their physicochemical characteristics, and their potential for ecotoxicity. The necessity for conducting risk assessment analysis arises from several factors, including the high solubility of the substance in water, its ability to persist in the environment, its tendency to accumulate in organisms, and its potential to induce toxicity and carcinogenicity. Indeed, this endeavor has a significant level of difficulty. Low concentrations of pharmaceutical environmental residues can potentially cause acute and chronic impacts on microorganisms, flora, and fauna. The observed effects encompass a spectrum of metabolic alterations and disruptions in hormonal equilibrium. Organisms other than the specified target species may experience adverse effects. Although present in tiny amounts, below the established threshold, certain pharmaceutical substances have the potential to inflict serious adverse effects due to the intricate interactions exhibited by diverse pharmaceutical mixes within the environment [6].

For the quantification of PhACs in water or soil sediments, various analytical biochemical methods have been utilized, including liquid chromatography-mass spectrometry (LC-MS), gas chromatography-MS (GC-MS), solid-phase extraction (SPE), hydrophilic interaction liquid chromatography (HILIC), and high-performance liquid chromatography-tandem mass spectrometry (HPLC-MS/MS) [53,54]. Nevertheless, in recent decades, technological advances in molecular biotechnology have improved the measurement and monitoring of pharmaceutical compounds’ ecotoxicological effects on water quality by applying and validating new biological indicators, such as bioassays and biomarkers [8,55,56,57,58,59,60,61,62].

The impact of human activities on different ecosystems is widely recognized, resulting in significant changes that include species extinction and biodiversity alterations. Cardinale et al. [63] highlighted that these changes can negatively affect ecosystem functioning. Hence, there is a demand for non-invasive assessments of biodiversity. According to Shim et al. [64], all living organisms release genetic material into the environment via various means, such as feces, urine, gametes, and epidermal cells, leaving detectable remnants of their DNA. In this context, biotechnological techniques based on next-generation sequencing (NGS), such as environmental DNA (eDNA) metabarcoding, can serve as a powerful bioindicator for detecting and evaluating the impacts of various pollutants, such as pharmaceutical compounds, on the diversity and composition of bacterial communities and other microorganisms such as microalgae (phytoplankton), protista, and metazoa [65,66,67,68,69]. Such techniques may also prove helpful for estimating the composition of animal and plant communities, including the genetic diversity of these species and their response to disease outbreaks resulting from changes in pathogen fitness and genotype–environment interactions due to the presence of specific PhACs [70]. The technique of eDNA metabarcoding entails an in-depth, thorough analysis of DNA sequences derived from environmental samples within a particular ecosystem [62,71,72,73,74]. The novel concept of eDNA metabarcoding, which offers to bypass many of the problems of thorough conventional research, is gaining traction as an effective and powerful approach to measuring biodiversity, albeit with pros and cons.

This review aims to provide an extensive overview of the emerging concept of pharmaceutical environmental pollution, focusing on the infiltration pathways of several PhACs into aquatic environments. Considering the ecotoxicological impacts of PhACs on organisms, this study highlights the significance of utilizing eDNA metabarcoding as a robust bioindicator method for evaluating these effects. More specifically, two primary facets were examined. Firstly, the issue of aquatic pharmaceutical pollution: the different categories of pharmaceutical pollutants were reviewed, emphasizing the characteristics, sources, fate, treatment methods, and impacts on the aquatic environment. Secondly, the importance of using eDNA metabarcoding as a biomonitoring tool to evaluate pharmaceutical environmental effects more consistently and effectively: the use of aquatic species as bioindicators to evaluate the implications of pharmaceutical pollution and the application of eDNA metabarcoding as a surveillance method of altered microbial communities, invertebrates, plants, and fishes were analyzed, thus contributing to more robust monitoring approaches and improved risk assessments. The eDNA metabarcoding methodology is presented comprehensively, encompassing technical details and analyzing its advantages and disadvantages.

The present study conducted a thorough examination of the existing literature using the established PRISMA principles [75]. A comprehensive literature review was performed using various search phrases in the ScienceDirect, Scopus, and PubMed databases. The inclusion criteria were restricted to studies published in English in peer-reviewed journals. The main focus was on research conducted between 2010 and 2023. Nevertheless, efforts have been undertaken to incorporate all relevant and significant reviews and full-length original articles that substantially contribute to the field, irrespective of the year of publication. The titles, abstracts, and keywords were subjected to a thorough examination to eliminate items that were not relevant to the study. The significance of this review is made evident by the author’s endeavor to incorporate, analyze, enhance understanding, and emphasize all the pertinent yet diverse and emerging research findings about the field of environmental pollution, biomonitoring, and eDNA metabarcoding.

## 2. Pharmaceuticals and Pollution: Routes and Pathways

**Figure 1:**
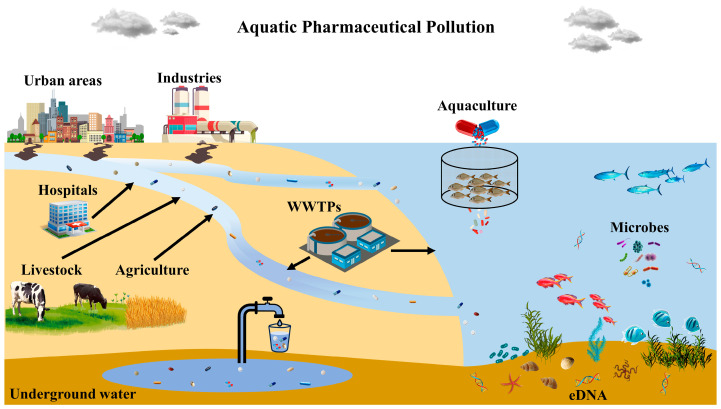
Main pathways of pharmaceutical aquatic pollution (WWTPs: wastewater treatment plants).

Many PhACs and byproducts exist in rivers, lakes, and groundwater [26]. Due to their widespread use, persistence, and bioaccumulation potential, several classes of PhACs have been identified as hazards to human and environmental health. They primarily infiltrate waterways through wastewater treatment plants, improper drug disposal, and human and animal waste (Figure 1).

PhACs can alter aquatic systems’ nutrient cycling, energy transmission, and microbial community composition, resulting in altered reproduction and development, as well as changing the behavior and survivability of almost all aquatic vertebrates and invertebrates [50,76]. Moreover, low antibiotic concentrations are associated with the survival and spread of antibiotic-resistant bacteria (ARBs) and antibiotic resistance genes (ARGs), which endanger human and animal health. Indeed, chronic exposure to trace amounts of PhACs in potable water or the consumption of contaminated aquatic organisms may result in medication resistance. To reduce PhACs’ contamination of water bodies and soil, upgrading treatment facilities or implementing new treatment methods is imperative. Depending on persistence, bioaccumulation, and exposure, pharmaceutical pollution varies by region and water body. Nevertheless, it is important to note that while conventional pollution typically has a more detrimental impact on hosts or pathogens when present in higher quantities, emerging pollutants such as pharmaceuticals can often exert their effects even at lower doses, usually after long-term exposure [6,77,78]. This phenomenon can be attributed to the specific manner in which these chemicals target conserved pathways [79]. The sensitization of the public, proper use and disposal, waste management, and purification have been proposed as essential measures to reduce pharmaceutical pollution and its potential adverse health and environmental effects [80]. Table 1 summarizes the main sources and the corresponding effects of these PhACs.

### 2.1. Antibiotics

Antibiotics are frequently employed in human as well as animal medicine for the purpose of treating bacterial infections. Penicillins, cephalosporins, lincosamides, macrolides, tetracyclines, sulfonamides, and quinolones are among the most frequently utilized classes of antibiotics in human medicine [89]. After ingestion, humans and animals frequently excrete antibiotics. Antibiotics and their constituents can also be released from untreated or inadequately treated effluents if conventional wastewater treatment methods are ineffective at removing them [35,36]. The excessive utilization of agricultural practices, such as farming and raising livestock, may be another source that contributes to the release of antibiotics into the surrounding environment.

Due to their vast utilization, the discharge of antibiotic-containing effluent into rivers, lakes, or other water bodies contributes significantly to pharmaceutical pollution [81,84,87,88,91,116]. Despite utilizing advanced treatment methods like activated carbon adsorption, ozone treatment, or other advanced methods, completely eradicating antibiotic residues from enriched wastes may not be achievable. Furthermore, the persistence of antibiotics in the environment can be attributed to their resistance to degradation [86]. Hence, antibiotics in the environment can potentially contribute to developing and spreading antibiotic resistance in microorganisms, posing significant challenges in treating infections. It can also exert selective pressure by disturbing the equilibrium of microbial communities in water bodies, thereby affecting nutrient cycling and the overall ecosystem functioning. This disturbance contributes to the emergence and dissemination of antibiotic-resistant bacteria (ARBs) and antibiotic resistance genes (ARGs) [82,83].

### 2.2. Hormones and Endocrine-Disrupting Chemicals

Endocrine-disrupting chemicals (EDCs) are naturally occurring or artificially produced compounds that interfere with the normal functioning of hormones in the body. Hormones such as estrogen are integral endocrine system components [117]. According to the United States Environmental Protection Agency (EPA), an EDC is an exogenous substance that possesses the capacity to interfere with the synthesis, secretion, transport, metabolism, receptor binding, or clearance of endogenous hormones, thereby inducing modifications in the endocrine and homeostatic systems [118,119]. EDCs are commonly found in a variety of everyday products, such as human and animal medications (e.g., diethylstilbestrol), cosmetics (e.g., triclosan), food and beverage packaging (e.g., perfluorochemicals, bisphenol A, phthalates), toys (e.g., lead and cadmium), industrial solvents or oils and their by-products (e.g., dioxins and polychlorinated biphenyls), and pesticides (e.g., dichlorodiphenyltrichloroethane and chlorpyrifos) [120,121,122]. EDCs can be classified into four distinct groups based on their source: industrial (e.g., dioxins, polychlorinated biphenyls, and alkylphenols), agricultural (including pesticides, insecticides, herbicides, phytoestrogens, and fungicides), residential (such as phthalates, polybrominated biphenyls, and bisphenol A), and pharmaceutical (including birth control pills, hormone replacement therapy, and parabens) [118,123,124].

Pharmaceutical EDCs can enter the environment through excretion and improper disposal [92,96,98]. Since hormones regulate human and animal physiological processes, their release into the environment through agricultural and livestock manure runoff can contribute to environmental pollution and ultimately disrupt the endocrine systems of aquatic organisms, resulting in reproductive and developmental abnormalities, altered sex ratios, and stunted growth and development [93,94,95,97,99,100]. Estrogenic hormones, such as estradiol and ethinyl estradiol (i.e., a synthetic estrogen present in contraceptive medications), are of special concern. The presence of these hormones has been linked to the occurrence of feminization effects in fish populations. Exposure to these hormones can induce the development of intersex traits, characterized by both male and female characteristics within a single individual, as well as the disturbance of normal reproductive processes. Fish feminization can have significant implications for population dynamics and reproductive success. It can sometimes lead to population declines or the extinction of endangered species [125,126].

Although the direct impact of hormone-contaminated water on human health is not yet fully understood, scientists continue to investigate the potential risks since it is believed that chronic exposure to low levels of hormone contaminants in potable water or consuming contaminated aquatic organisms may subtly affect human endocrine systems [127].

### 2.3. Analgesics and Nonsteroidal Anti-Inflammatory Drugs

Analgesics and nonsteroidal anti-inflammatory drugs (NSAIDs) are commonly employed to manage pain and inflammation. The detection of analgesics and NSAIDs in both ground and surface water, such as lakes and rivers, has become prevalent due to their extensive utilization. Minute amounts of NSAIDs have been identified in various environmental matrices such as soil, wastewater, surface water, groundwater, sediments, snow, and drinking water [39,104]. Despite negligible detectable environmental concentrations, NSAIDs have long-lasting ecotoxic impacts on the biotic components of ecosystems [103,104]. According to Feng et al. [128], daily NSAID consumption exceeds 30 million doses and is rising swiftly. Due to their stability and resistance to degradation, these compounds can persist in the environment and accumulate over time. NSAIDs can enter the environment through various routes, but human excretion is the most common. The inappropriate disposal of unused medications further contributes to NSAID pollution [129]. Additionally, pharmaceutical manufacturing and healthcare facility effluent discharges can release NSAIDs into the environment. NSAIDs can influence organisms’ behavior, reproduction, growth, and development. For instance, NSAIDs such as ibuprofen and diclofenac have been associated with impaired reproduction and aberrant development in fish. In addition, repeated exposure to NSAIDs may cause bioaccumulation in aquatic organisms. Bioaccumulation can occur through the food chain, leading to elevated levels of NSAIDs in predators that consume contaminated prey. NSAIDs can influence the microbial communities of aquatic ecosystems by inhibiting the development and activity of beneficial bacteria, resulting in population imbalances among microorganisms and disruption of essential ecological processes [101,102].

### 2.4. Psychotropic and Antiepileptic Drugs

Psychotropic medications and AEDs are commonly employed in managing mental health disorders, such as anxiety and depression, addiction, seizures and convulsions, and chronic pain management. Researchers have discovered traces of psychotropics and AEDs in aquatic environments, implying their widespread presence. Psychotropic and AED drugs infiltrate the environment primarily through human excretion following their use as medications. The active compounds of psychotropic medications are metabolized within the body, and the residues are excreted through urine and feces. Also, these drugs can infiltrate wastewater systems via sewage or septic tanks [43,44,108]. Studies have demonstrated that exposure to psychotropic medications can induce alterations in the behavior, reproductive patterns, and physiological functions of fish, invertebrates, and other aquatic organisms [106,107], contributing to further disrupting the natural ecological processes and aquatic ecosystem populations.

### 2.5. β-Blockers

β-blockers are a class of pharmaceutical drugs (competitive antagonists) that inhibit the activity of adrenergic β-receptors in the sympathetic nervous system. The broad range of pathologies for which β-blockers are prescribed has resulted in an annual consumption increase of more than treble [130,131]. Although β-blockers have significant therapeutic value, they can potentially contribute to pharmaceutical pollution and have environmental effects. The increased consumption of β-blockers has led to increased tracing in the environment, and their presence has been detected in several bodies of water [132,133]. The administration of β-blockers has the potential to exert adverse effects on fish, as evidenced by their ability to induce alterations in pulse rate and other cardiovascular-related physiological processes. They have been found to have various impacts on aquatic organisms, such as causing disruption in testosterone levels, reducing fertility and reproduction rates, and inducing abnormal behavior [109,110,111]. β-blockers can alter the activities and functions of microorganisms involved in decomposing organic matter during treatment within wastewater treatment facilities [112].

### 2.6. Chemotherapy and Anticancer Drugs

Chemotherapy and anticancer medications may pose risks as pharmaceutical pollutants when discharged into the environment [115]. Human excretion is the primary source after administering these medications to cancer patients. However, the incorrect disposal of unused medicines can contribute to chemotherapy drug pollution. Typically, conventional wastewater purification processes are ineffective at removing chemotherapy drugs. Thus, these drugs can enter the environment through treated effluents [114,134]. Certain chemotherapy drugs are designed to be exceedingly robust and resistant to degradation to exert their therapeutic effects on the human body. This stability will also allow them to endure for extended periods in the environment and, consequently, may accumulate over time, resulting in long-term exposure in particular regions [114,135]. Chemotherapeutic medications may adversely affect aquatic organisms and other non-target species [113]. They can affect the growth, development, and reproduction of organisms exposed to them by interfering with normal cell division and DNA replication. Several investigations have demonstrated toxic effects at environmental concentrations on fish, invertebrates, and other aquatic organisms [114]. Additionally, chemotherapy drugs can disrupt the growth and activity of soil and water microbial communities in the environment, leading to population imbalances and disturbances in ecological processes.

## 3. Treatment Methods for Pharmaceutical Pollution

Mitigating pharmaceutical contamination necessitates a comprehensive strategy encompassing regulatory measures, appropriate disposal practices, and effective treatment methodologies. Traditional water treatment facilities employ a multifaceted approach encompassing several physical, chemical, and/or biological techniques to enhance water quality (Table 2). However, it is important to note that most treatment approaches exhibit certain drawbacks, including secondary pollution, elevated maintenance expenses, and intricate procedures involved in the treatment process [136]. Conventional treatment techniques, such as chlorination, filtration, and coagulation–flocculation, exhibit limited efficacy in eliminating pharmaceuticals [137]. The ineffectiveness of these technologies in maintaining appropriate levels of water safety and quality has become evident due to the increasing presence of pharmaceuticals in environmental waters. Hence, there is a pressing need to develop more efficient and sophisticated water treatment technologies to mitigate the potential risks associated with pharmaceuticals in water. These technologies should integrate traditional methods’ strengths while incorporating novel and innovative solutions [138,139]. Several studies have provided evidence that various treatment approaches, including membrane bioreactors [140], bacterial or fungal treatments [141,142], adsorption, nanofiltration, and reverse osmosis, have proven to be successful in the removal of contaminants [143,144,145]. In recent times, the utilization of electrochemical oxidation, in conjunction with other advanced oxidation processes (AOP), has emerged as an up-and-coming method for eliminating pharmaceuticals from water and wastewater [146]. AOPs encompass the production of highly reactive species that can break down or fully mineralize specific chemical contaminants, even when present in minute concentrations. Over the past few decades, extensive research has been conducted on various AOPs for water and wastewater remediation. These processes encompass photolytic, chemical, photochemical, physical, and photocatalytic mechanisms [147]. Among these, the photocatalytic process is of particular significance, with the choice of photocatalyst being a crucial factor. Notably, TiO_2_ has been widely employed for degrading pharmaceutical compounds and other chemical pollutants [148,149,150,151]. Table 2 below provides comparative information on the efficiency of different treatment methods for removing major pharmaceutical compounds from water and wastewater.

## 4. Methods of Analysis, Detecting, and Monitoring of Pharmaceutical Pollution

For the analysis and quantification of EPPPs and PhACs in aquatic environments, numerous sophisticated chromatographic and spectroscopic methodologies and instrumentation, such as LC-MS, GC-MS, SPE, HILIC, and HPLC-MS, are commonly employed, which have allowed for the high-throughput monitoring of hundreds of chemicals even at exceedingly low quantities [53,54,195,196,197,198,199,200,201,202,203,204,205,206,207,208,209,210]. HPLC is widely recognized as the predominant analytical technique in the field. This method is employed to analyze diverse environmental pollutants, such as PhACs, which typically exhibit polarity and instability across various sample types. The utilization of LC techniques has been found to offer an effective stationary phase through particle size reduction. This reduction in the particle size leads to improved fixation and a decrease in the duration of the process [211]. Consequently, in the majority of instances, ultra-high-performance liquid chromatography (UHPLC) has been employed instead of conventional HPLC. Also, GC is a widely employed analytical technique to classify, analyze, and identify chemical constituents in diverse samples. When used in conjunction with MS, the GC method is considered the most systematic approach, as it can produce precise and reliable results. GC is preferred over LC for determining the most polar contaminants, such as those found in pharmaceuticals [212]. Due to the high polarity and low flexibility of analytes such as hydroxyl, phenolic endocrine-disrupting chemicals, amines, and amides, the utilization of alternative output is required in the GC method to enhance the chromatographic behavior of analysts [213].

Although the chemical analysis of the environment matrix is the most straightforward method to uncover the presence of pharmaceutical pollution in the environment, this approach alone may not provide compelling evidence regarding the comprehensive impact and potential toxicity of such pollution on organisms and the ecosystem as a whole.

### 4.1. Bio-Monitoring and Pharmaceutical Surveillance Methods

The risk characterization of aquatic ecosystems is highly important and involves assessing potential damage to freshwater and/or marine organisms and the effects on humans [214]. The significance of biomonitoring studies in aquatic ecosystems lies in its role in evaluating the response of these ecosystems to disruptions and in unraveling the intricate relationships among physical, chemical, and biological factors [215]. These studies are essential for assessing aquatic ecosystem well-being since organisms excel as indicators of environmental conditions, frequently providing insights beyond what conventional water quality measurements can reveal [216,217,218,219].

The integration of field and traditional laboratory investigations is of paramount importance in the field of ecotoxicology. However, relying only on either approach may only sometimes yield comprehensive results in terms of identifying and quantifying the potential ecological risk posed by chemical stressors. The evaluation of the detrimental impacts of pollutants on ecosystem processes can be conducted through the utilization of experimental systems, such as sediment [220] and stream microcosms or mesocosms [221,222]. In addition, novel approaches exist for evaluating ecological well-being, including stressor-specific indicators like pollution-induced community tolerance (PICT) [223], multivariate diagnostic tools for assessing the responses of microbial communities to pollutants [224], and the SPEcies AT Risk index (SPEAR) for detecting the adverse effects of toxic stress on macroinvertebrate communities [225]. These methods prove to be valuable in assessing ecological health. Furthermore, the evaluation of chemicals might extend beyond their direct harmful effects on individual species, encompassing their potential indirect impacts on community structure [226], population dynamics, and ecosystem services [227,228]. It has been demonstrated that community ecology models (e.g., food web modeling) are effective for evaluating ecologically significant adverse effects in aquatic ecosystems [229]. Yet, ecotoxicologists are still wondering how to safeguard all biodiversity from the variety of chemicals they are now exposed to, when we know relatively little about real-world exposures and even less about the flora and fauna that we want to protect [230].

#### 4.1.1. Bioindicator Species: Methods and Platforms

Scientists utilize biological markers to detect environmental contamination, ranging from plants and animals to microorganisms [231]. It is common practice to use microorganisms, primarily bacteria, as markers of the overall health of both marine and terrestrial ecosystems. Bacterial pharmaceutical pollution indicators specifically include measuring the composition of the entire microbial community, quantifying several sole bacterial species (e.g., Escherichia, Pseudomonas, Acinetobacter, Rhodococcus spp., etc.), or groups (e.g., bioluminescent, or nitrifying bacteria), and assessing the abundance in selective ARBs communities [232]. In polluted environments, measuring the abundance of specific genetic (e.g., ARGs) or protein bacterial indicators (e.g., metabolic biochemical markers) is also employed to assist in identifying particular pharmaceutical pollutants and their consequences [233].

Several effect-based methods and technological interventions can be used in monitoring routine and investigative endeavors to assess the ecological condition related to pollution burden [234,235,236]. Microbial biosensors (cell-free and whole-cell-based) [237,238,239] metagenomic and metatranscriptomic methods, such as high-throughput sequencing of organisms exposed to chemicals [240,241,242,243], have the potential to improve the efficacy of structure-based ecosystem analyses. These approaches can establish more explicit links between chemicals, their modes of action, and ecological functions [244]. Table 3 outlines examples of bacteria indicators coupled to different experimental sets and platforms associated with several types of pharmaceutical pollution (directly or indirectly).

#### 4.1.2. eDNA Metabarcoding

Over the past few years, eDNA metabarcoding has gained widespread popularity as a means to assess the ecological effects of pharmaceutical pollution on natural aquatic ecosystems by unveiling the phylogenetic diversity of specific species (e.g., [269,270,271,272]), as well as on the structures and co-occurrence patterns of multi-trophic communities [273,274,275]. eDNA refers to the genetic material obtained from various environmental sources, such as soil, water, and air, without the requirement of isolating specific target organisms beforehand [276]. It has a wide temporal persistence range, spanning from a few weeks to many thousands of years, thus permitting its utilization in various fields such as molecular biology, ecology, paleontology, and environmental sciences [277]. In contemporary times, the methodologies employed in eDNA investigation, specifically eDNA metabarcoding, have made significant progress, enabling the evaluation of entire ecological communities through the analysis of a solitary sample. This is achieved by utilizing high-throughput NGS techniques to discern the species composition within the sample [277].

Using eDNA analysis to detect and quantify the biodiversity of micro- and macro-organisms enables the research community to study an ecosystem without requiring physical capture or visual surveys [278]. Consequently, it can address the limitations of other labor-intensive conventional methods and investigate the presence of organisms at a location by identifying eDNA in environmental samples [279]. Pont et al. [280] suggested a quantitative approach to aquatic community analysis using eDNA methods, which appeared more suitable for biomonitoring and bioassessment purposes than other traditional methods. By combining qPCR analysis and eDNA metabarcoding, using 12S rRNA, they allowed for the estimation of species diversity and abundance in the Danube River, overall detecting 86 fish taxa [280]. Similarly, Yang et al. [281] proposed an unsupervised biological assessment framework based on multi-gene eDNA metabarcoding for the health evaluation of Lake Taihu. This framework could allow consistent evaluation across various ecosystems and seasons, thereby supporting environmental management and decision-making. They were able to describe a total of 478 species using 18S rRNA, while COI and 12S rRNA identified 99 and 66 species, respectively, including algae, protists, zooplankton, and fish. In their study, the limitations of traditional supervised assessment methods and the need for more standardized indicators and assessment methods were highlighted, emphasizing the potential of eDNA technology for efficient and non-invasive biomonitoring [281].

eDNA metabarcoding can serve as a practical and highly sensitive tool for biodiversity monitoring [282], offering several key advantages in aquatic research [283,284]. It can effectively identify a wide range of freshwater and marine species, often outperforming traditional survey methods in species detection [285,286,287]. It can detect rare and cryptic species often missed by conventional survey techniques, including endangered and invasive species [288,289,290]. eDNA collection requires only tiny amounts of water and basic filtering techniques [291,292], making it accessible even in remote locations by individuals with limited training. eDNA eliminates the need for diving, enhancing worker safety [284,293], and it is cost-effective and has the potential for automation [294,295], enabling remote sample collection and efficient high-throughput lab processing. Lastly, the complex disruptions arising from a combination of natural and human-induced factors, as well as the increasing rate of biodiversity decline and the diminishing ecological function in aquatic ecosystems, reinforce the need for using eDNA metabarcoding as a dependable approach for assessing the influence of pollutants, e.g., pharmaceuticals, on aquatic organisms [66,275,296].

The experimental procedure steps for the development of eDNA metabarcoding surveys have been extensively described in various studies [277,297,298] and comprise a selection of specific gene(s) and primers for targeting particular taxa [299,300], the compilation or creation of extensive barcode reference databases [299,301], the implementation of stringent decontamination pipelines based on site occupancy (e.g., [302,303]), initial investigations performance to characterize spatial and temporal variations in eDNA (e.g., [304,305,306]) and the storage of samples, extracts, and raw sequence data for future reference (e.g., [307,308,309]).

Even though the application of eDNA metabarcoding has grown enormously, there are also concerns regarding its strengths and limitations. The effectiveness of this method in aquatic environments depends on its ability to detect species even at low abundance levels, as well as cryptic, rare, or elusive organisms [310]. A key point in ecological assessments and biodiversity monitoring is sensitivity, as it enables researchers to reveal the hidden aspects of aquatic ecosystems and track changes in species composition over time [311,312]. Various factors, such as sample collection methods, DNA extraction protocols, and the choice of genetic markers, which play a crucial role in maximizing the method’s detection capabilities, need to be taken into consideration to reach the desirable levels of sensitivity in eDNA metabarcoding [312].

Another crucial point for the application of this method is accuracy, as it directly affects the reliability of species identification and community assessments within aquatic environments [311,313]. Ensuring precision and reducing the risk of false positive and negative identifications in ecological data is essential in species detection [314]. Optimal accuracy depends on the proper selection of genetic markers, the utilization of suitable primer sets, robust bioinformatic pipelines for data analysis, and updated databases for correct species recognition [315]. Precise eDNA metabarcoding enhances our comprehension of aquatic ecosystem biodiversity and establishes a valid basis for informed decisions in conservation and management [316].

As with any other method, it may not work, or when it does, it might not provide the requisite information. Challenges relating to imperfect detection, quantifying abundance, assigning taxonomies, understanding the spatial and temporal dynamics of eDNA, analyzing and interpreting data, and assessing ecological conditions have all proven to be significant hurdles [279,317]. These challenges and limitations of eDNA metabarcoding have been the subject of various biodiversity and monitoring studies [314,318,319]. However, before its adoption, researchers have focused on addressing and overcoming the disadvantages of the method (e.g., [320,321,322]).

Overall, this method provides a powerful supplement or alternative to traditional survey methods in measuring and monitoring the biodiversity and health of aquatic ecosystems at unprecedented resolution and scale [66,311,323,324,325]. Nowadays, it constitutes one of the primary surveys employed by researchers and public agencies towards ecosystem conservation and meeting many resource management issues across nations [322,326,327].

## 5. Environmental Impact of Different Sources of Pharmaceutical Pollution

Industrialization, urbanization, and economic development damage rivers, lakes, and oceans [328]. Nutrient pollution can induce toxic algal overgrowth, fish deaths, waterborne disease outbreaks, and eutrophication, which pollutes and depletes oxygen [329,330]. The increasing use of pharmaceuticals and their persistent occurrence in aquatic environments significantly impact various species across different taxonomic levels [331]. These effects extend from microbial communities and aquatic plants to macro-invertebrates, fishes, and humans. In the following sections, the main implications of pharmaceuticals on the different aquatic organisms were described, as well as the utilization of eDNA metabarcoding as a reliable methodology for examining the impacts of pollutants, specifically pharmaceuticals, on aquatic ecosystems [332,333,334,335]. Table 4 summarizes the main implications of PhACs and the role of eDNA metabarcoding as a bio-monitoring surveillance method for environmental/pharmaceutical pollution. In the following Section 5.1, Section 5.2 and Section 5.3, we elaborate on the effects of pharmaceutical pollution and the potential role of eDNA metabarcoding in evaluating these effects.

### 5.1. Alteration of Microbial Communities Due to Pharmaceutical Contamination

Multiple research studies have investigated the impact of pharmaceutical substances on microbial communities in aquatic environments. The synthesis of these studies reveals that pharmaceutical contaminants can induce alterations in the structure, metabolic activity, composition, and formation of microbial biofilms [5,336]. These modifications can potentially impact the equilibrium of nutrients in surface waters, soil, and marine ecosystems and contribute to microbiological concerns in potable water. Furthermore, the primary emphasis in studying the influence of pharmaceuticals on microbial communities in the environment lies in investigating the consequences of minimal levels of antibiotics. At the same time, attention is also given to other emerging contaminants that are not antibiotics, such as NSAIDs. These emerging contaminants have the potential to exert selective pressure and facilitate the proliferation of antimicrobial resistance [336].

The primary purpose of antibiotics is to combat pathogenic bacteria. Nonetheless, the potential impact of byproducts and residues on non-target species, such as algae and cyanobacteria, which play a crucial role as primary producers in aquatic ecosystems, cannot be overlooked [338,377]. The perturbations in these photoautotrophic organisms’ populations can significantly impact higher trophic levels. The literature has extensively examined the toxic effects of antibiotics as individual pharmaceuticals, as well as their biodegradation products, in diverse aquatic environments [14,35,339,340]. Through the utilization of various bioassays, researchers have demonstrated that most microorganisms exhibit susceptibility to prolonged exposure to varying concentrations of antibiotics, with cyanobacteria emerging as the most probable candidate.

This fortuitous encounter suggests that the administration of antibiotics has the potential to disrupt the balance of microbial populations, leading to an increase in ARBs and ARGs. In recent years, there has been a notable emergence of ARBs and ARGs as environmental contaminants with the capacity for rapid global dissemination [90]. ARGs, along with mobile genetic elements (MGEs) such as plasmids, integrons, and transposons, have the ability to disseminate through horizontal gene transfer. This process is facilitated by three mechanisms: transformation, conjugation, and transduction [85].

DNA-based techniques, e.g., polymerase chain reaction (PCR) and quantitative PCR (qPCR), are required to investigate the possible transmission of ARBs and ARGs within microbial communities, as well as their transfer to higher organisms. In the past decade, by utilizing these techniques, researchers have demonstrated the presence of bacteria and the existence of genes associated with resistance to different antibiotics and antimicrobial drugs in samples from various water sources, such as water treatment facilities [378,379,380,381], residential areas [379,382], hospitals [379], lakes [383], rivers [384,385], and aquaculture facilities [386]. These findings indicate that regions characterized by human exploitation significantly contribute to the spread of microbial antibiotic resistance.

In addition to conventional DNA-based methodologies such as PCR and qPCR, which allow for the targeted detection of particular microbial species, the application of metabarcoding techniques has facilitated the assessment of changes in the composition and diversity of microbial communities resulting from specific PhACs [342,345]. Examining microbial communities’ structure and identifying potential hazards, such as bacterial contamination in water sources, are of utmost importance. In this regard, Cruz et al. [343] employed the sequencing of the 16S rRNA gene amplicon to analyze the DNA of bacterial communities in diverse water sources, including treated and untreated hospital wastewater, fish culture sites, lakes, and urban waste canals, situated in Sri Lanka and the Philippines. The bacterial communities’ composition and abundance were found to be influenced by the water source, with untreated wastewater samples exhibiting higher bacterial richness. This finding underscores the significance of comprehending bacterial communities in assessing water quality.

Romero et al. [344] utilized 16S rRNA metabarcoding to examine microbiota in various sub-basins of the Rimac River, which serves as the primary water source for Lima, Peru. The investigation focused on areas with persistent multidirectional water pollution and aimed to compare the diversity patterns between the Andean and Metropolitan regions. The higher prevalence of bacteria in samples collected from lower altitudes and the high occurrence of *Arcobacter cryaerophilus*, a pathogen associated with fecal contamination and antibiotic resistance, underscores the necessity for utilizing NGS techniques to augment pathogen surveillance. In a similar investigation, Chonova et al. [333] effectively employed metabarcoding to analyze diatom communities’ spatial and temporal dynamics. The study aimed to evaluate these communities’ ecological responses, growth patterns, and behavioral tendencies in environments containing diverse pharmaceutical pollutants, including β-blockers, NSAIDs, and antibiotics.

Other recent reports also suggest that eDNA methods could provide valuable insights into predicting pollution levels and identifying the key factors influencing ecological networks. Li et al. [66] employed eDNA metabarcoding to effectively characterize a diverse array of bacteria and ascertain the primary source of contamination in rivers, encompassing multiple anthropogenic pollutants such as excessive nutrients, heavy metals, pesticides, and pharmaceuticals. In another study, Lyu et al. [275] identified the occurrence of 91 antibiotics in water and sediment samples obtained from the Nei River. Additionally, they utilized eDNA metabarcoding to investigate the observed fluctuations in bacterial communities. Significant inverse associations were observed between antibiotic concentrations and the relative abundances of vital metabolic pathways in bacterial populations.

Furthermore, the increase in urbanization and land use poses a significant threat to freshwater ecosystems due to the release of various chemical contaminants [387], which can affect organismal dispersal and nutrient transport in water [388,389]. Xie et al. [341], by examining freshwater sediments from the Nanfei River in Anhui Province, China, suggested that eDNA metabarcoding on in situ eukaryotic communities can be a valuable method for biomonitoring and detecting chemical pollution originating from diverse land use types, such as agricultural and industrial areas. All these findings highlight how eDNA analysis is an effective tool for providing comprehensive data regarding the influence of pharmaceutical pollutants on aquatic microbial diversity.

### 5.2. Effects of Pharmaceuticals on Aquatic Invertebrates, Plants, and Fishes

Despite typically occurring at low concentrations in aquatic environments, pharmaceutical compounds exhibit considerable biological activity, often coupled with remarkable stability [390]. As a result, there is increasing concern about their potential ecotoxicological effects on aquatic fauna and flora, especially over extended periods of exposure [34]. To develop a comprehensive understanding of the potential risks that pharmaceuticals may pose to aquatic life, it is crucial to assess their prevalence across various organisms, including plants and algae, invertebrates, and fish [53].

Photosynthetic organisms, including phytoplankton and macrophytes, constitute a significant proportion of the overall biomass present in aquatic ecosystems [347]. Primary producers release oxygen and constitute key carbon sources, nutrients, and trace elements [391]. They also provide food and shelter for many aquatic species, affecting water flow patterns and reducing sediment erosion [347]. Accumulating emerging contaminants in water bodies (e.g., pesticides) has been shown to induce harmful effects during plant development [346] and impair aquatic plant photosynthesis and biodiversity [3]. Among various PhACs, antibiotics have an antichloroplastic activity in cyanobacteria, green algae, and other aquatic plants, primarily due to the similarity of their target sites for toxic action with those of bacteria [347]. However, micro- and macrophytes could provide important environmental services by bioremediating pollutants in natural environments [392]. They are equipped with multiple detoxification mechanisms that aid in mitigating the deleterious effects of pollutants and counteracting the toxicity of various exogenous substances [393]. Following Bala et al. [394], contaminants can undergo partial or complete degradation, broad metabolism, or be transformed into less toxic compounds. These transformed compounds can then be incorporated into plant tissues in a form that cannot be easily extracted [395].

The role of aquatic invertebrates (e.g., sponges, corals, worms, echinoderms, crustaceans, and shellfish) in nutrient cycling, processing substantial amounts of organic matter, and serving as a food source for numerous organisms makes them crucial for the functioning of aquatic ecosystems [396]. The existing literature indicates that pharmaceutical pollution can notably affect macroinvertebrates, mainly in terms of growth, behavior, and reproduction [357]. However, the absorption and bioaccumulation of pharmaceuticals in these organisms are subject to considerable variability, contingent upon habitat conditions (both abiotic and biotic factors) and their physiological attributes, encompassing chemical and biological elements [397]. The task of making reliable inferences about the bioaccumulation of pharmaceuticals in invertebrates is considerably more complex, primarily attributable to the inconsistent presence of pharmaceutical compounds in benthic species across diverse sampling sites [398]. Furthermore, the bioaccumulation process is intricately linked to the species’ type, distribution, and abundance, which can vary spatially and temporally within aquatic ecosystems [399]. 

Relative to the effects of PhACs in aquatic vertebrates, extensive research has been conducted on fish, primarily due to their role as a reliable indicator of aquatic pollution. This is attributed to their ability to accumulate contaminants from the surrounding water and their vulnerability to experiencing adverse effects [358]. The predominant chronic adverse impacts of pharmaceuticals on fish species primarily manifest as locomotor and reproductive dysfunctions, hematological and hormonal imbalances, immunotoxicity, disruption of endocrine function, genotoxicity, oxidative stress, physical deformities, teratogenic effects, and a deterioration in the overall physiological state of the organisms [39,358,359,360,361]. The initial exposure of fish to xenobiotics in aquatic environments occurs via their gills, leading to potential structural impairments and physiological changes in these tissues [400,401]. However, the degree of consequences of pharmaceutical substances in the various health aspects of fish depends on species, sex, and the phase of the life cycle, as well as on the dose and duration of the substances [361,402,403]. Furthermore, the presence of pharmaceuticals in aquatic environments has also been observed to influence the behavior of fish, comprising alterations in activity, sociality, and feeding rate, as well as aggression and reproductive behaviors [362,363,364,365].

Identifying species abundance and diversity changes can provide valuable insights into the ecological consequences of pharmaceutical exposure. In aquatic ecosystems, conventional biotic indices, such as morphological identification, are insufficient to accurately represent the actual population structure, since species in these environments may remain concealed underwater, and their detection through traditional means could be challenging [348,350]. Therefore, the utilization of eDNA in ecological research, biomonitoring, and environmental management can be beneficial and bring about a transformative impact on the field of conservation science [404]. Several studies have reported the use of eDNA analysis to investigate the biotic composition, abundance, and distribution of aquatic species, such as macroinvertebrates, plants, fishes, and others in freshwater and marine ecosystems [348,349,350,351,352,353,354].

Fish comprise more than one quarter of the world’s vertebrate species and are also one of the most threatened taxonomic groups [405]. They are the most frequently targeted taxa in species-specific metabarcoding studies, probably due to their economic importance [406]. Alterations in fish composition and abundance as bioindicators of aquatic systems subjected to anthropogenic stressors suggest that eDNA technology could be used to revolutionize fish monitoring, foster biodiversity, conservation, and fishery management that transcends both geographical and temporal boundaries [355,356].

Although eDNA surveys for groups like fishes are being standardized, this differs for other aquatic taxa such as macroinvertebrates [407] and plants [408]. Humanization processes like water pollution and habitat fragmentation lead to a decline in macroinvertebrate populations, highlighting the importance of rapid, precise, and homogeneous eDNA monitoring for species survival [409,410,411]. Following Pawlowski et al. [412] and Pochon et al. [413], the application of metabarcoding techniques has shown that fish farming significantly impacts benthic foraminifera communities, indicating alterations in species richness near aquaculture facilities. However, they mentioned the need for the replication of samples and the interpretation of read abundance data. To overcome these biases and limitations, more experimental studies are needed to improve the accuracy and reliability of NGS metabarcoding as well as the taxonomic assignment of NGS reads, enhancing the effectiveness of NGS metabarcoding as a biomonitoring tool for macroinvertebrates [412]. Finally, the absence of single universal plant barcodes hinders the use of metabarcoding for studying contemporary marine plants. However, recent studies show promising results in assessing plant biodiversity monitoring in aquatic ecosystems [408]. Overall, the necessity for sensitive biomonitoring tools to support conservation initiatives aimed at safeguarding these susceptible organisms is paramount.

### 5.3. Bioaccumulation and Trophic Transfer of Pharmaceuticals in Aquatic Food Webs

The bioaccumulation of pharmaceuticals is a critical concern, potentially with significant implications for aquatic life and human health. As these pharmaceutical compounds enter aquatic ecosystems through various pathways, they can be taken up and stored in the tissues of aquatic organisms [366,367]. Subsequently, these contaminants can be transferred through the food web, leading to higher concentrations in higher trophic levels (namely biomagnification), including fish and macroinvertebrates [368]. Given the widespread consumption and cost-effectiveness of aquatic organisms (mainly fish) as a protein source in various global regions, it is plausible that pharmaceutical substances could be introduced into the human body through trophic transfer, potentially posing risks to human health [358]. Due to their complex nature, food webs cannot be easily understood by merely considering the sum of their constituent elements (e.g., fish play a significant role within aquatic food webs, moving between trophic levels during their ontogeny) [414], the understanding of the dynamics of trophic transfer and bioaccumulation of pharmaceuticals in different aquatic species is essential for assessing and mitigating potential health hazards associated with these emerging contaminants [367].

Pharmaceuticals are being detected more frequently in environmental samples, yet our understanding of their movement through aquatic food webs, known as trophic transfer, still needs to be fully resolved [367]. Using conventional methods, scientists have already studied the bioaccumulation of such substances at various trophic levels [415,416]. In this field, however, multiple challenges need to be addressed. A comprehensive understanding of ecology is essential to gain insights into the accumulation and dispersion of pharmaceuticals in aquatic food webs [417]. The successful management and preservation of ecosystems necessitate a comprehensive worldwide endeavor to consistently monitor the biological communities’ composition and diversity [418]. Current ecological assessment methods are constrained by their reliance on traditional morpho-taxonomic approaches, the assumption of environmental sorting of communities, and the identification of species by proficient analysts, methods that cannot keep up with the growing need for swift assessments [369].

eDNA data for biomonitoring provide distinct advantages over current methods as they may detect a broader spectrum of taxa and indicator groups that traditional taxonomic identification may miss, leading to more accurate assessments, particularly when comparing nearby locations or evaluating moderate environmental changes [369]. Its use in ecotoxicology has also become a crucial advancement in recent years, allowing for the detection and quantification of the effects of toxic substances on ecological communities [370]. DNA metabarcoding is being used for various applications like identifying individual species and assessing community compositions in aquatic ecosystems [369], investigating biodiversity [313,373], characterizing prey in gut contents or fecal samples [374,375], and analyzing food web dynamics [376]. It can also provide valuable insights into the impacts of chemical stressors on freshwater ecosystems and allows for identifying keystone species and monitoring shifts in microbial functional groups, which can help predict potential changes in ecosystem functionality [317]. Anagnostopoulos et al. [371] used metabarcoding analysis to assess bacterial communities and potential pathogens in water and fish flesh sampled from various locations within Lake Karla (Eastern mainland Greece). This approach offered a comprehensive view of the microbial composition and diversity within the samples, revealing the impact of agricultural and industrial activities on both water quality and fish safety. Li et al. [372] utilized DNA metabarcoding to assess the impact of paroxetine (SSRI antidepressants) on multi-trophic microorganisms and nitrogen transformation in river sediments. This study underscores the significance of this approach in offering valuable insights into the composition and dynamics of microbial communities responding to the tested pharmaceutical [372]. Despite the numerous potential benefits of eDNA-based assessment, such as enhanced sensitivity, broader spatial and temporal coverage, and reduced personnel demands [348,419], it still requires validation as a reliable alternative to existing biomonitoring protocols [369].

## 6. Future Perspectives

Ecosystems worldwide are changing as they enter a new geological era in which human interventions (e.g., climate change, habitat destruction, environmental pollution) dramatically affect the environment [420,421]. Among the various global environmental challenges, a notable rise of contaminants, including pharmaceuticals, personal care products, pesticides, and microplastics, has been noted in aquatic ecosystems worldwide over the past few decades [6]. Environmental pharmaceutical pollution has garnered international attention over the past two decades [422] in light of significant global issues such as rising antimicrobial resistance and the lack of new antibiotic molecules [5]. Pharmaceuticals can negatively affect aquatic ecosystems and wildlife. However, the complete scope of ecological impacts must be comprehended, including the short- and long-term effects on various species and habitats. It is anticipated that pharmaceutical pollution will continue to rise exponentially and globally due to population growth, aging populations, and increased access to healthcare, all of which will increase pharmaceutical consumption and its potential release into the environment.

Global pharmaceutical pollution necessitates developing and implementing effective strategies for monitoring, mitigating, and preventing pharmaceutical pollution by multiple parties [423]. Governments and non-government organizations are attempting to combat the pollution caused by pharmaceuticals (e.g., Environmental Risk Assessment–ERA) [14]. The overall scheme of these proposed—but not yet fully managed and implemented—efforts includes the ethical use of antibiotics, which refers to the responsible administration of antibiotics in human and veterinary medicine, the understanding of the path that these medications take in the environment and how they are transported, the raising of awareness about the negative effects that pharmaceuticals have on the environment, the promoting of the safe disposal of unused prescription drugs, and the supporting of the safe disposal of unused pharmaceuticals. These combined efforts and approaches aim to reduce the pollution caused by a broad range of pharmaceutical products and limit the potential impact of some PhACs on the surrounding environment [8,424].

The utilization of sophisticated molecular biology techniques and traditional biochemical methods enables us to efficiently degrade or accumulate harmful substances from the environment, thereby mitigating pharmaceutical pollution. Microorganisms and plants possessing biosynthetic pathways for the degradation or accumulation of environmental pollutants in soil and water have the potential to mitigate environmental pollution, including that induced by pharmaceutical compounds. Nevertheless, the limited presence of distinct genetic elements in microorganisms and plants hampers their ability to break down or accumulate pollutants effectively. In recent years, significant progress has been made in the field of CRISPR-Cas9 technology, which has facilitated the manipulation of genetic material in microorganisms and plants. This has been employed to enhance the effectiveness of reducing the degradation and accumulation of environmental pollutants [425,426]. Although welcomed and alleviative, these approaches are far from efficient enough, and more holistic approaches are needed.

Hence, the observation (biomonitoring) and understanding the dynamics and shifts (biodiversity) of various types of species’ pollution remain crucial, as well as the need for more innovative solutions to address pharmaceutical pollution effectively. To achieve this objective, biomonitoring can be carried out qualitatively through observing and documenting alterations in organisms or quantitatively by assessing the accumulation of compounds within the tissues of organisms. Through the process of observing or measuring the impacts of the environment on native organisms, it becomes possible to raise suspicions or make inferences about pollution. Consequently, appropriate actions can be prioritized based on these findings [284].

eDNA metabarcoding shows great potential as a novel strategy for comprehensively assessing and monitoring aquatic ecosystems [66,404]. The application of metabarcoding in studying the impacts of pharmaceuticals on aquatic ecosystems has been demonstrated as an effective and valuable methodology [427]. Certain medications can alter microbial communities in soil, water, and detritus, and metabarcoding can assist in identifying these alterations, thereby serving as an early warning system for pharmaceutical contamination. Thus, through metabarcoding and high-throughput NGS, eDNA can rapidly, repeatedly, and affordably survey community biodiversity [283]. By integrating eDNA metabarcoding with other complementary methodologies, such as biochemical analysis and conventional ecotoxicological assessments, scientists can synergistically gain insight into the ecological consequences of pharmaceutical pollution and design effective management strategies. In addition, metabarcoding can be used to monitor the effectiveness of pharmaceutical pollution mitigation measures. By comparing the composition and diversity of microbial communities before and after the implementation of interventions, such as improved effluent treatment or regulatory measures, researchers can evaluate the effectiveness of these interventions. Furthermore, metabarcoding enables the detection of emergent contaminants that may not be included in standard monitoring programs. It allows for the identification of unknown or novel pharmaceuticals that may have contaminated the environment and aids in prioritizing research and regulatory efforts to combat these emergent threats. 

Despite the several benefits of species detection and community monitoring that have been mentioned, several important considerations need to be made before employing eDNA techniques [428]. The accuracy of the assessment is significantly dependent on the sampled material type. eDNA detection is typically more efficient in aquatic environments than in detritus or soil [429]. The sample’s quality and quantity are also crucial factors. This aspect is closely related to the quantity of DNA released into the environment by each species. For instance, species such as fish and amphibians tend to discharge substantial amounts of DNA [316,430]. In addition, certain habitats, particularly those that are difficult to access, hinder species tracking more than others [431]. Consequently, the assessment of species assembly in aquatic environments was more effective in small, stationary freshwater habitats, such as lakes and ponds, than in large, moving waterways, such as streams and rivers [432]. Lastly, accurate species identification is impacted by species abundance within the studied medium and eDNA changes over time and space [73,433]. Nonetheless, obstacles, including PCR inhibition, eDNA capture, and representative sampling, impede the discovery of complete species diversity in aquatic environments. In addition to the specificity of primers and the quality of the reference database, the level of taxonomic expertise influences the success of species identification. Measuring species abundance, associating species detections with the actual species composition of the ecosystem and determining species interaction are additional challenges in implementing eDNA-based approaches [279].

## 7. Conclusions

In conclusion, to effectively manage pharmaceutical pollution, governments, regulatory bodies, pharmaceutical producers, healthcare professionals, scientists, and the general public must cooperate in an efficient and productive manner. By implementing the methods mentioned above, we may make strides in lowering the environmental and health risks of pharmaceutical pollution and promoting a more sustainable approach to the consumption of medications and their disposal. Despite any limitations and obstacles, the novel idea of eDNA metabarcoding, which bypasses many of the difficulties associated with conducting extensive conventional environmental research, is gaining traction as a means of measuring and monitoring biodiversity alterations due to pharmaceutical pollution, indicating the presence of pharmaceuticals, assessing ecological impacts, and tracking the efficacy of mitigation efforts. These benefits further advance our knowledge of the scope and effects of pharmaceutical pollution and help us make informed further decisions about mitigating its negative impact on the environment. Harnessing technological innovations, eDNA metabarcoding emerges as a highly promising approach for assessing communities across a spectrum of applications, spanning from ecosystem restoration to human health, underscoring its pivotal role in the future of molecular research.

## Figures and Tables

**Table 1:** Overview of the primary sources and associated environmental effects and health risks of PhACs.

Pharmaceuticals	Sources	Effects	References
Antibiotics	Contamination of water bodies from human and veterinary medicine wastes.	Induce an increase in ARBs and (ARGs); Disrupt the equilibrium of natural microbial communities in water bodies; Affect the cycling of nutrients and the overall functioning of the ecosystems; Cause potential long-term effects on human health.	[81,82,83,84,85,86,87,88,89,90,91]
Hormones and endocrine-disrupting chemicals (EDCs)	Enter the aquatic environment through agricultural and livestock manure, excretion (e.g., urine and feces), improper disposal.	Contribute to hormone pollution and ultimately disrupt the endocrine systems of aquatic organisms; Induce developmental abnormalities in fish; Alter sex ratios and reproductive success in fish populations; Affect the growth and development of aquatic organisms; Affect human endocrine systems by chronic exposure to low levels of hormone contaminants in potable water or consuming contaminated aquatic organisms.	[92,93,94,95,96,97,98,99,100]
Analgesics and nonsteroidal anti-inflammatory drugs (NSAIDs)	Main pathways to the aquatic environment: human excretion, the inappropriate disposal of unused medications, wastewater discharges from pharmaceutical and healthcare facilities.	Induce long-lasting ecotoxic effects on the biotic components of ecosystems; Induce detrimental effects on plants, including growth inhibition, cellular and root damage, and metabolic disorders; Influence the behavior, reproduction, growth, and development of fish, amphibians, and invertebrates; Cause bioaccumulation in aquatic predators through the food chain; Affect the microbial communities of aquatic ecosystems and disrupt essential ecological processes.	[27,101,102,103,104,105]
Psychotropic and antiepileptic drugs	Enter water bodies through human excretion, and wastewater systems via sewage or septic tanks	Inhibit the growth of aquatic organisms; Alter fish, invertebrates, and other aquatic organisms’ behavior, reproduction, and physiological functions; Disrupt natural ecological processes and aquatic ecosystem populations.	[43,44,106,107,108]
β-blockers	Infiltrate the aquatic environment through human excretion, and wastewater systems via sewage or septic tanks	Alter fish pulse rate and other cardiovascular-related physiological processes; Induce testosterone disruption, decrease fertility, reproduction rates, and aberrant behavior in aquatic organisms; Affect microbial communities within wastewater treatment facilities.	[109,110,111,112]
Chemotherapy and anticancer drugs	Introduction to the aquatic environment via human excretion and the incorrect disposal of unused medicines	Affect organisms’ growth, development, and reproduction by interfering with normal cell division and DNA replication; Induce toxic effects at environmental concentrations on fish, invertebrates, and other aquatic organisms; Disrupt microbial communities in the environment, including soil and water microbial communities, through alterations in the growth and activity of beneficial microbes, leading to microbial population imbalances and disturbances in ecological processes.	[113,114,115]

**Table 2:** Removal efficiency of different treatment methods for pharmaceutical compounds (based on and modified by Stadlmair et al. [152]).

Type of Treatment Method	Treatment Method	Efficiency	Pharmaceutical Compounds	References
Physico-chemical Treatment	Aeration	Low	Analgesics and antibiotics	[153]
	Coagulation, flocculation, and sedimentation	Very low	Antibiotics, antidepressants, AEDs, analgesics, NSAIDs	[154,155,156]
	Adsorption	High	Antibiotics and NSAIDs	[157,158,159,160,161]
	Filtration	Contaminant dependent	AEDs, NSAIDs, antibiotics, EDCs	[162,163,164,165]
	Nanofiltration	Moderate to high	EDCs, β-blockers, psychotropics and AEDs, antibiotics	[166,167,168,169]
	Reverse osmosis	High	Analgesics, NSAIDs, β-blockers, AEDs, psychotropic drugs	[170,171,172]
Biological Treatment	Conventional activated sludge	Low to moderate	Analgesics, EDCs, antibiotics, β-blockers, AEDs	[173,174]
	Membrane bioreactors	Moderate to high	EDCs, psychotropic drugs, NSAIDs, anti-diabetic drugs, β-blockers,	[108,140,175]
	Microalgal bioremediation process	Low to moderate	NSAIDs, β-blockers, AEDs, antibiotics, EDCs	[176,177]
	Enzyme-based treatment	Moderate to high	NSAIDs	[152,178,179,180]
Oxidation Treatment	Chlorination	Contaminant-dependent	Antibiotics, EDCs, β-blockers, analgesics, NSAIDs	[181,182]
	Ozonation	High	Antibiotics, EDCs, AEDs, NSAIDs, psychotropic drugs	[172,183,184,185]
	Advanced oxidation processes (AOPs)	High	EDCs, antibiotics, NSAIDs, psychotropic drugs	[89,186,187,188,189]
Electrochemical treatment	Electrochemical technologies	High	Antibiotics, EDCs, NSAIDs	[190,191,192,193,194]

**Table 3:** Summary of bacterial indicators and coupled methods.

Methods Associated with the Use of Bacteria for Pharmaceutical Pollution		Indicative Taxa (or Genus or Phylum)	Pharmaceutical Compounds	Method of Detection	Refs.
Indirect Methods	Bioremediation	Actinobacteria, *Chryseobacterium*, *Flavobacterium*, *Pseudoxanthomonas*,	β-blockers	PCR-DGGE and pyrosequencing	[245]
		*Bacillus thuringiensis* B1 *Novosphingobium* sp. *Sphingomonas* sp. *Sphingopyxis* sp. *Sphingobium* sp. *Isoptericola* sp. *Nubsella* sp. *Rhodococcus* sp. *Bacillus* sp.	NSAIDs	Gram staining, API CORYNE system analysis, FAMEs analysis, HPLC, cell cultures, PCR	[246,247]
		*Pseudomonas* sp. CE21 *Pseudomonas* sp. CE22 *Paucibacter* *Filomicrobium*	Antibiotics	Cell cultures, PCR, LC-MS, degradation analysis with MS, BOD 5/COD Ratio, HPLC, TOC/TN analysis, ammonia and nitrate analysis, SEM	[248,249]
		*Chryseobacterium taeanense* *Rhizobium daejeonense* *Diaphorobacter nitroreducens* *Achromobacter mucicolens* *Pseudomonas veronii* *Pseudomonas lini*	AEDs	PCR and HPLC	[250]
		*Microbacterium* sp. C448	Anti-cancer	Liquid scintillation counting, HPLC-MS/MS, LC-MS, solvent extraction (ASE 200), NGS	[251]
		*Flavobacterium* *Novosphingobium* sp. *Sphingomonas* sp. *Sphingopyxis* sp. *Sphingobium* sp. *Isoptericola* sp. *Nubsella* sp. *Rhodococcus* sp. *Bacillus* sp. *Nitrosomonas europaea* *Acinetobacter* sp. *Phyllobacterium myrsinacearum* *Ralstonia pickettii* *Pseudomonas*	EDCs	HPLC, IC, TOC analysis, oxygen probe analysis, rep-PCR, NGS, fluorescence detection, colorimetric analysis, UV/fluorescence detection, GC-MS/MS and LC-MS/MS, ATP/OD measurement	[247,252,253,254]
	Communities’ function and structure	Actinobacteria, Bacteroidetes, Cyanobacteria, Flavobacteria, Firmicutes, Proteobacteria, *Fusobacteria*	Hormones, antibiotics, antipsychotic drugs, AEDs, NSAIDs, β-blockers, antihistamines, antidiabetics, analgesics, H_2_ blockers, ACE inhibitors	HPLC, UPLC-MS/MS, FTIR, LC-MS/MS, enzyme assays, MBR and batch cultures, qPCR, PCR-DGGE, NGS, metagenomics	[255,256,257,258,259]
	Detection of ARBs (and ARGs)	*Escherichia coli*, *Klebsiella pneumoniae*, *Aeromonas* spp., *Pseudomonas aeruginosa*, *Enterococcus faecalis*, *Enterococcus faecium*, *Acinetobacter baumannii*, *Flavobacterium*, *Poriferibacter*, *Bacteroides*, *Acinetobacter*, *Actinobaculum*, *Streptococcus*	Antibiotics	Metagenomics-metatranscriptomics, qPCR, rep-PCR	[260,261,262]
Direct Methods	Whole-cell Biosensors	*Escherichia coli*, *Pseudomonas fluorescen*, *Bacillus subtilis*	Antibiotics, NSAIDs, EDCs	Biosensor (optical, fluorescence, electrochemical, etc.)	[263,264,265,266,267,268]

PCR: polymerase chain reaction, qPCR: quantitative polymerase chain reaction, rep-PCR: repetitive extragenic palindromic polymerase chain reaction, DGGE: denaturing gradient gel electrophoresis, FAMEs: fatty acid methyl ester, HPLC: high-performance liquid chromatography, LC-MS: liquid chromatography–mass spectrometry, MS: mass spectrometry, BOD: biochemical oxygen demand, COD: chemical oxygen demand, TOC: total organic carbon, TN: total water-born nitrogen, SEM: scanning electron microscopy, NGS: next generation sequencing, IC: ion chromatography, GC-MS/MS: gas chromatography tandem mass spectrometry, LC-MS/MS: liquid chromatography tandem mass spectrometry, ATP: adenosine triphosphate, OD: optical density, UPLC-MS/MS: ultra performance liquid chromatography tandem mass spectrometry, FTIR: Fourier transform infrared spectroscopy, MBR: membrane bioreactors.

**Table 4:** Overview of the role of eDNA metabarcoding in assessing aquatic organisms’ response to pharmaceutical pollution.

Affected Aquatic Organisms	Summary of PhACs Implications	Evaluation of eDNA Metabarcoding in Pharmaceutical Pollution Assessment
Microbial communities	Alterations in the structure, metabolic activity, composition, and formation of microbial biofilms [5,336]; Disruption of the balance of microbial populations, leading to an increase in ARBs and ARGs [90,336]; Perturbation in micro-photoautotrophic organisms which can impact higher trophic levels [14,35,337,338,339,340].	Provides comprehensive data regarding the influence of pharmaceutical pollutants on aquatic microbial diversity [341]; Facilitates the assessment of changes in the composition and diversity of microbial communities resulting from specific PhACs [342,343,344,345]; Provides valuable insights into predicting pollution levels and identifying the key factors influencing ecological networks [66,275].
Plants	Harmful effects during plants’ development [346]; Antichloroplastic activity in cyanobacteria, green algae, and other aquatic plants [347]; Perturbation in macro-photoautotrophic organisms which can significantly impact higher trophic levels [14,35,337,339].	Assesses the biotic composition, abundance, and distribution of aquatic species (i.e., macroinvertebrates, plants, fishes, and others in freshwater and marine ecosystems) [348,349,350,351,352,353,354]; Improves fish monitoring, fosters biodiversity conservation and fishery management that transcends both geographical and temporal boundaries [355,356].
Invertebrates	Notable effects on macroinvertebrates, mainly in terms of growth, behavior, and reproduction [357].	
Vertebrates	Chronic adverse impacts on fish species that manifest mainly as locomotor and reproductive dysfunctions, hematological and hormonal imbalances, immunotoxicity, the disruption of endocrine function, genotoxicity, oxidative stress, physical deformities, teratogenic effects, and a deterioration in the overall physiological state of the organisms [39,358,359,360,361]; Influences on the behavior of fish, comprising alterations in activity, sociality, and feeding rate as well as aggression and reproductive behaviors [362,363,364,365].	
Aquatic food webs	Bioaccumulation: pharmaceutical compounds enter aquatic ecosystems and can be stored in the tissues of aquatic organisms [366,367]; Biomagnification: contaminants can be transferred across the food web, leading to higher concentrations in the aquatic organism tissues of higher trophic levels [368], potentially posing risks to human health [358].	Detects a broader spectrum of taxa and indicator groups that traditional taxonomic identification may miss, leading to more accurate assessments [369]; Provides valuable insights into the impacts of chemical stressors on freshwater ecosystems and allows for identifying keystone species and monitoring shifts in microbial functional groups, which can help predict potential changes in ecosystem functionality [317,370,371,372]; Identifies individual species and assesses community compositions in aquatic ecosystems [369], investigates biodiversity [313,373], characterizes prey in gut contents or fecal samples [374,375], and analyzes food web dynamics [376].

## Data Availability

Not applicable.
